# Aortic insufficiency following transcatheter aortic valve replacement is underestimated by echocardiography compared with cardiac MRI

**DOI:** 10.1186/1532-429X-16-S1-O101

**Published:** 2014-01-16

**Authors:** Wissam M Abdallah, Chris A Semder, Evan L Brittain, Michael T Baker, Lisa A Mendes, Marshall H Crenshaw, Joseph L Fredi, Mark A Robbins, Sonia L Scalf, William S Bradham, Sean G Hughes, Mark A Lawson, David X Zhao

**Affiliations:** 1 Division of Cardiovascular Medicine, Vanderbilt University Medical Center, Nashville, Tennessee, USA; 2 Division of Cardiology, Wake Forest University, Winston-Salem, North Carolina, USA

## Background

The degree of aortic insufficiency (AI) after transcatheter aortic valve replacement (TAVR) has been identified as a predictor of increased mortality. Since even mild AI is associated with increased mortality in some studies, accurate quantification of post-TAVR AI is critical. Assessment of AI by echocardiography is typically performed by visual inspection and semi-quantitative methods. Most post-TAVR AI is paravalvular, however echocardiography has limited ability to quantify multiple eccentric paravalvular jets. Using flow quantification methods, cardiac MRI (CMR) may more accurately quantify AI severity post-TAVR and therefore more accurately assess risk in this population.

## Methods

Twenty-three patients who underwent TAVR with a SAPIEN prosthesis were studied. All patients underwent an intraoperative transesophageal echocardiogram (TEE), as well as a post-procedure transthoracic echocardiogram (TTE) and CMR. Paravalvular AI by TTE and TEE was graded using color Doppler by quantifying the circumferential extent of AI as a percentage of the aortic annulus (none < 1%, trace 1-5%, mild 6-10%, moderate 11-30%, severe >30%) following recommendations from the Valve Academic Research Consortium. AI severity by CMR was quantified as the regurgitant fraction of forward aortic flow based on previously published recommendations (none <1%, trace 1-5%, mild 6-15%, moderate 16-48%, severe >48%).

## Results

**Figure 1:**
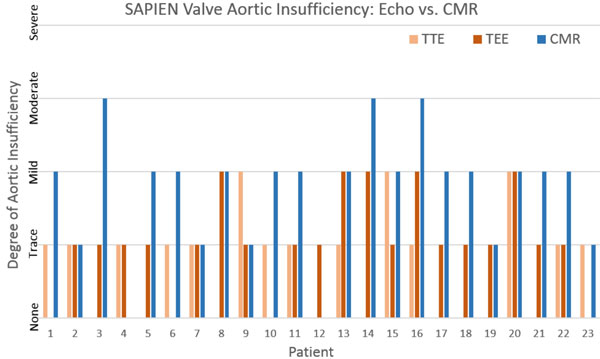


**Figure 2:**
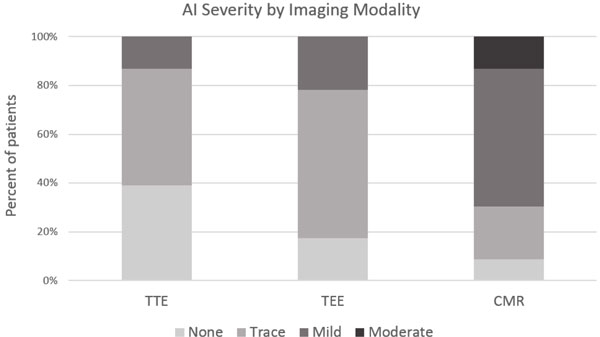


The mean age was 79 +/- 10 years; 52% were men. TTE and CMR were performed at 1 [1-1] and 4 [1-4] days post-TAVR respectively (median [IQR]). The left ventricular ejection fraction (LVEF) by CMR was 65 +/- 10%. AI severity by TTE was none in 9 (39.1%), trace in 11 (47.8%), and mild in 3 (13%) patients. TEE identified trace central AI in 6 patients (26%). Paravalvular AI by TEE was none in 4 (17.4%), trace in 14 (60.9%), and mild in 5 (21.7%) patients. AI by CMR was none in 2 (8.7%), trace in 5 (21.7%), mild in 13 (56.5%), and moderate in 3 (13%) patients; (Figure 1). A higher proportion of patients with mild or greater AI was identified by CMR (16/23, 70%) compared to TTE (3/23, 13%) and TEE (5/23, 22%); (Figure 2).

## Conclusions

CMR can reliably quantify post-TAVR AI. Echocardiography underestimates the severity of post-TAVR AI compared with CMR. CMR could be selectively utilized in patients with mild post-TAVR AI by echocardiography to screen for more significant AI. Further studies are needed to determine 1) whether echocardiography alone underestimates the risk of adverse outcomes related to post-TAVR AI, and 2) whether similar degrees of post-TAVR AI by CMR also translate into adverse outcomes.

## Funding

None.

